# Frontline Science: LPS‐inducible SLC30A1 drives human macrophage‐mediated zinc toxicity against intracellular *Escherichia coli*

**DOI:** 10.1002/JLB.2HI0420-160R

**Published:** 2020-05-22

**Authors:** Claudia J. Stocks, Jessica B. von Pein, James E.B. Curson, James Rae, Minh‐Duy Phan, Darren Foo, Nilesh J. Bokil, Taiho Kambe, Kate M. Peters, Robert G. Parton, Mark A. Schembri, Ronan Kapetanovic, Matthew J. Sweet

**Affiliations:** ^1^ Institute for Molecular Bioscience (IMB) The University of Queensland St. Lucia Queensland Australia; ^2^ IMB Centre for Inflammation and Disease Research The University of Queensland St. Lucia Queensland Australia; ^3^ Australian Infectious Diseases Research Centre The University of Queensland St. Lucia Queensland Australia; ^4^ School of Chemistry and Molecular Biosciences The University of Queensland St. Lucia Queensland Australia; ^5^ Division of Integrated Life Science, Graduate School of Biostudies Kyoto University Kyoto Japan; ^6^ Centre for Microscopy and Microanalysis The University of Queensland St. Lucia Queensland Australia

**Keywords:** antimicrobial, *E. coli*, host‐pathogen, metal ions, zinc toxicity, zinc transporters

## Abstract

TLR‐inducible zinc toxicity is an antimicrobial mechanism utilized by macrophages, however knowledge of molecular mechanisms mediating this response is limited. Here, we show that *E. coli* exposed to zinc stress within primary human macrophages reside in membrane‐bound vesicular compartments. Since SLC30A zinc exporters can deliver zinc into the lumen of vesicles, we examined LPS‐regulated mRNA expression of *Slc30a*/*SLC30A* family members in primary mouse and human macrophages. A number of these transporters were dynamically regulated in both cell populations. In human monocyte‐derived macrophages, LPS strongly up‐regulated SLC30A1 mRNA and protein expression. In contrast, *SLC30A1* was not LPS‐inducible in macrophage‐like PMA‐differentiated THP‐1 cells. We therefore ectopically expressed SLC30A1 in these cells, finding that this was sufficient to promote zinc‐containing vesicle formation. The response was similar to that observed following LPS stimulation. Ectopically expressed SLC30A1 localized to both the plasma membrane and intracellular zinc‐containing vesicles within LPS‐stimulated THP‐1 cells. Inducible overexpression of SLC30A1 in THP‐1 cells infected with the *Escherichia coli* K‐12 strain MG1655 augmented the zinc stress response of intracellular bacteria and promoted clearance. Furthermore, in THP‐1 cells infected with an MG1655 zinc stress reporter strain, all bacteria contained within SLC30A1‐positive compartments were subjected to zinc stress. Thus, SLC30A1 marks zinc‐containing compartments associated with TLR‐inducible zinc toxicity in human macrophages, and its ectopic over‐expression is sufficient to initiate this antimicrobial pathway in these cells. Finally, *SLC30A1* silencing did not compromise *E. coli* clearance by primary human macrophages, suggesting that other zinc exporters may also contribute to the zinc toxicity response.

## Graphical Abstract

The zinc transporter SLC30A1 is LPS‐inducible in human macrophages and can deliver a zinc toxicity response against intracellular *Escherichia coli*.

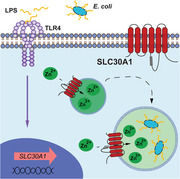

**Abbreviations**
BMM: bone marrow‐derived macrophages
CLEM: correlative light‐electron microscopy
EV: empty vector
HMDM: human monocyte‐derived macrophages
MCS: multiple cloning site
p.i.: post‐infection
qPCR: quantitative PCR

## 1 INTRODUCTION

macrophages and other innate immune cells utilize an array of antimicrobial mechanisms to defend against pathogenic bacteria.^1^ One such strategy, nutritional immunity, refers to the competition between host cells and invading microorganisms for essential nutrients and metal ions.^2^ For example, neutrophils secrete the zinc and manganese‐binding protein calprotectin to restrict availability of these critical trace elements to bacteria, thus limiting their growth and survival.^3^ Several pathogens counteract these and other innate immune responses by employing sophisticated mechanisms to acquire essential metal ions.^4^ For example, *Salmonella* uses the ZnuABC zinc uptake system for host colonization^5^ and to defend against innate immune‐mediated nitrosative stress.^6^

In recent years, it has become clear that specific trace elements such as zinc can also be harnessed by innate immune cells as antimicrobial agents to combat bacterial infection.^7^ In *Mycobacteria*‐infected macrophages exposed to inflammatory cytokines, phagosomal zinc concentrations were reported to reach millimolar concentrations.^8^ *Mycobacterium tuberculosis* upregulates heavy metal efflux P‐type ATPases in the intramacrophage environment, with an accumulation of zinc within bacteria‐containing phagosomes also being observed.^9^ Consistent with this, TLR‐mediated macrophage activation triggers the mobilization of zinc into vesicular‐like structures that co‐localize with engulfed *E. coli*, with intracellular bacteria producing a transcriptional response consistent with zinc poisoning.^10^ The zinc toxicity response is also deployed by the soil amoeba *Dictyostelium discoideum*, suggesting this is an ancient host defense pathway.^11^ In keeping with a central role of zinc poisoning in innate immune antimicrobial responses, the bacterial pathogens *Salmonella* ^10^ and uropathogenic *E. coli* ^12^ are able to both resist and evade this response. Mechanisms by which mobilized zinc exerts antimicrobial effects within innate immune cells are unknown. However, studies on bacteria alone have implicated induced copper deficiency,^13^ manganese deficiency resulting in increased sensitivity to oxidative stress,^14^, ^15^ the replacement of other cations in essential enzymes,^7^ and disruption of iron‐sulfur (4Fe‐S) biogenesis with consequential arrest of key metabolic pathways^16^, ^17^ as factors that contribute to the antimicrobial effect of zinc. Interestingly, zinc can synergize with reactive oxygen species to limit *E. coli* growth.^12^ Thus, zinc toxicity could also act in a combinatorial fashion with other innate immune antimicrobial pathways.

Despite existing knowledge of innate immune‐mediated zinc toxicity, we still have a very limited understanding of the molecular processes underpinning this response. Mammalian zinc homeostasis is tightly regulated by 2 families of transporters, the ZIP/SLC39A family that delivers zinc to the cytoplasm and the ZnT/SLC30A family that moves zinc from the cytoplasm to the extracellular space or to the lumen of organelles.^18^ SLC39A zinc importers likely contribute to the uptake and thus availability of intracellular zinc for the zinc toxicity pathway. However, macrophage‐expressed SLC39A transporters have mainly been studied in the context of regulation of inflammatory responses. For example, *SLC39A8* is up‐regulated in human monocytes and macrophages in response to either TNF or LPS,^19^ with this being important for control of inflammatory cytokine production.^20^ SLC39A8 functions as a negative regulator of NF‐κB, as zinc can directly bind and inhibit IκB kinase.^19^ Some studies have also investigated the roles of SLC30A zinc exporters in innate immune functional responses. LPS up‐regulates expression of *Slc30a1*, *Slc30a4*, and *Slc30a6* in murine dendritic cells, with regulated zinc trafficking linked to antigen presentation.^21^ GM‐CSF increased mRNA levels of *Slc30a4* and *Slc30a7* in murine peritoneal macrophages, with the subsequent sequestration of zinc in the Golgi apparatus being associated with impaired survival of the intracellular fungal pathogen *Histoplasma capsulatum*.^22^ In contrast, while IL‐4 stimulation of bone marrow murine macrophages similarly induced *Slc30a4* expression, this induction promotes a SLC30A4‐dependent increase in intracellular zinc that favors survival of *H. capsulatum*,^23^ suggesting complex roles for individual zinc transporters in host defense. In this study, we focused on the SLC30A zinc exporter family and demonstrate that SLC30A1 is sufficient to deliver a zinc toxicity response against intramacrophage bacteria.

## 2 MATERIALS AND METHODS

Extended methods describing bacterial strains, correlative light‐electron microscopy (CLEM), infection assays, lentiviral construct generation, immunoblotting, flow cytometry, quantitative real time PCR (qPCR), and selective gene silencing can be found in the Supplementary material.

### 2.1 Ethics statement

All work involving primary human cells was approved by The University of Queensland Medical Research Ethics Committee (Certificate number 2013001519). Work involving mice was approved by the University of Queensland Animal Ethics Committee (Approval number IMB/123/18).

### 2.2 Mammalian cell culture

To generate human monocyte‐derived macrophages (HMDM), CD14^+^ monocytes were isolated from human buffy coats (Australian Red Cross Blood Service) and cultured for 7 days with 150 ng/ml recombinant human CSF‐1 (University of Queensland Protein Expression Facility), as previously described.^12^ To generate mouse bone marrow‐derived macrophages (BMM), bone marrow cells from the femurs and tibias of C57BL/6 mice were cultured for 7 days as previously described.^12^ Human monocytic THP‐1 cells were obtained from the American Tissue Culture Collection. Non‐adherent THP‐1 cells were differentiated into macrophages in presence of 30 ng/ml PMA (Sigma‐Aldrich) for 48 h.

### 2.3 Inducible gene expression in THP‐1 cells

The lentiviral system utilizing pF_TRE3G_PGK_puro (hereafter pLenti_EV: kindly provided by James Murphy, Walter and Elizabeth Hall Institute of Medical Research) for doxycycline‐inducible gene expression has previously been described.^24^, ^25^ Details on specific lentiviral expression constructs can be found in the Supplementary material and Supplementary Table 1. pLenti_MCS (Empty Vector) or pLenti_SLC30A1_V5 was introduction into THP‐1 cells by lentiviral transduction, as previously described.^25^, ^26^ Briefly, Lipofectamine 2000 (Invitrogen) in OptiMEM was combined with pCMV‐dR8.2 dvpr and pCMV_VSV‐G (Addgene), along with the appropriate DNA transfer plasmid (pLenti_EV or pLenti_SLC30A1_V5). Viral particles were harvested at 24 and 48 h post‐transfection and applied to non‐differentiated THP‐1 cells via a 2 h, 1000 × *g* spinfection at 35°C. Cells were subsequently maintained in 1 μg/ml puromycin (Sigma‐Aldrich). Inducible overexpression of SLC30A1 was confirmed through treatment with 100 ng/ml doxycycline (Sigma‐Aldrich) for 24 h, with subsequent experiments proceeding as described.

### 2.4 Confocal microscopy

Immunofluoresence imaging of fixed cells was performed using a Zeiss Axiovert 200 Upright Microscope Stand with LSM 710 Meta Confocal Scanner. Cells grown on coverslips were washed twice with PBS and then fixed in 4% PFA for 10 min. Nuclear DNA was stained with 1 μg/ml DAPI (Life Technologies). Intracellular zinc was detected by incubating fixed cells with 5 μM FluoZin‐3 AM (Life Technologies), as previously described.^10^ Lentivirally transduced THP‐1 cells were washed with PBS, permeabilized, and blocked with 0.1% BSA, before being incubated with anti‐V5 and anti‐mouse Alexa‐647 Abs in 0.1% BSA, respectively (see Supplementary Table 2 for concentrations). DAPI, GFP, mCherry, and Alexa‐647 were excited with laser emission of 405, 488, 561, and 647 nm, respectively. Blinded, unbiased quantification was performed through examination of images (minimum 65 cells per experiment) via Image J, with cells scored for the presence/absence of zinc puncta or the presence and number of fluorescent bacteria.

### 2.5 Statistical analyses

Individual experiments were typically performed in experimental duplicate or triplicate, with mean values taken from each experiment for combining data from at least 3 experiments for statistical analyses. Prism 5 and/or 7 software was used to perform the specific statistical tests that are indicated in the individual figure legends.

## 3 RESULTS AND DISCUSSION

### 3.1 Zinc‐stressed *E. coli* reside within membrane‐bound compartments in human macrophages

**FIGURE 1:**
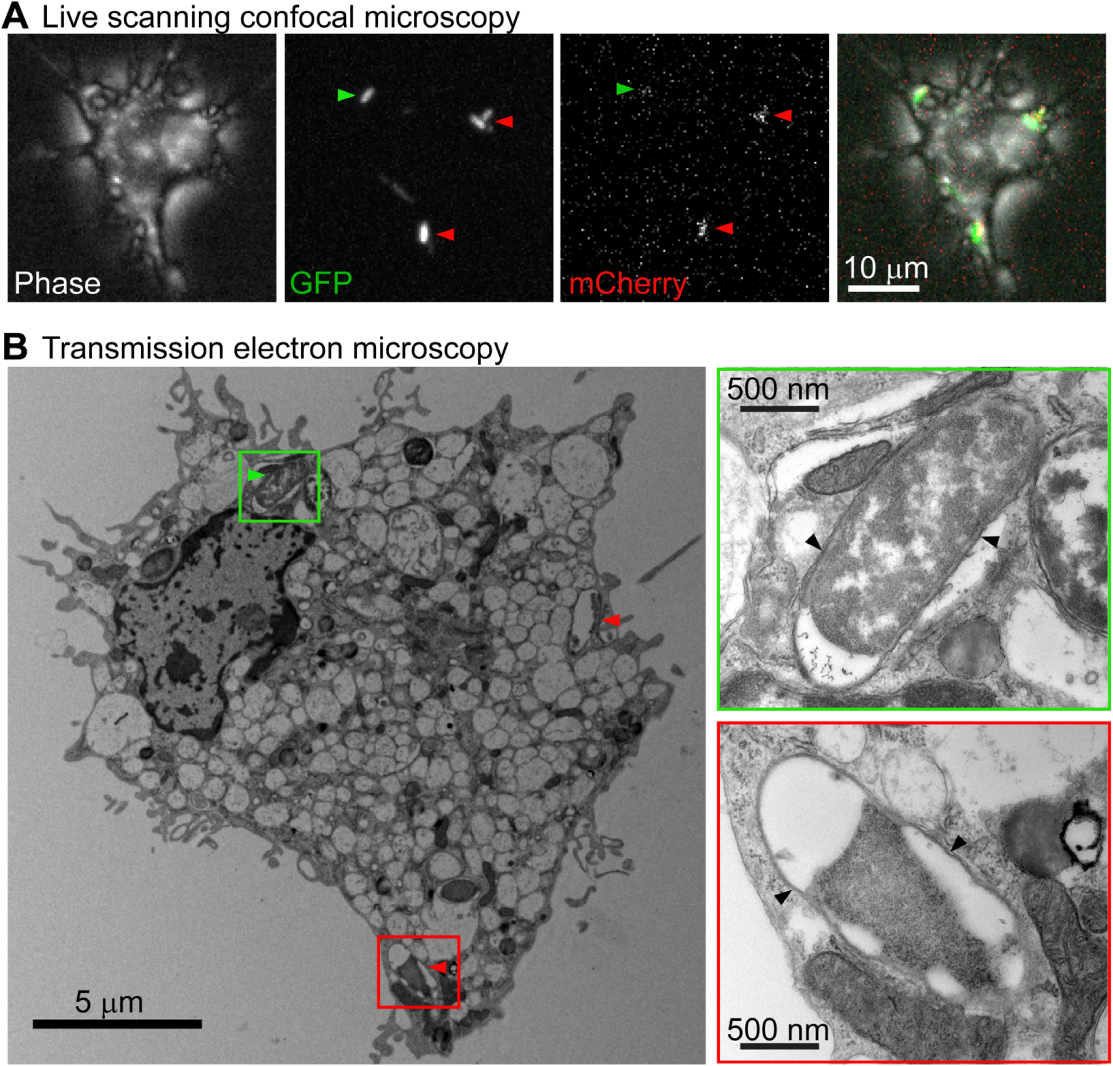
**Zinc‐stressed *E. coli* resides within membrane‐bound compartments in infected human macrophages**. HMDM were infected with MG1655 pGcCzntAp (MOI 100) for 1 h, followed by gentamicin exclusion. (**A**) At 8 h p.i., live cells were imaged via phase contrast and for GFP and mCherry fluorescence. (**B**) Cells were fixed in 2.5% glutaraldehyde, embedded, and sectioned, then imaged by transmission electron microscopy. Green arrows indicate GFP^+ve^ only bacteria, while red arrows indicate GFP^+ve^, mCherry^+ve^ bacteria. In both cases, bacteria indicated in (**A**) correlate with putative bacteria indicated in (**B**) and are shown in a green or red inset, respectively. Black arrows indicate visible membrane surrounding bacteria. Images are of a single cell and are representative of 3 cells from 1 experiment

*E. coli zntA* encodes a zinc efflux system that confers zinc resistance, as originally demonstrated through transposon mutagenesis, targeted gene deletion and gene complementation studies.^27^, ^28^ We previously showed that the *E. coli* K‐12 strain MG1655 is exposed to zinc stress during macrophage infection, as evidenced by intramacrophage expression of zinc‐inducible *zntA* and reduced intramacrophage survival of a *zntA* mutant strain.^10^, ^12^ To track this antimicrobial response, we also developed a zinc stress reporter strain (MG1655 pGcCzntAp), which constitutively expresses GFP and inducibly expresses mCherry when bacteria are subjected to zinc stress.^12^ To further investigate this phenomenon, we utilized CLEM to visualize MG1655 pGcCzntAp within HMDM (Fig. 1). Live imaging of this zinc stress reporter strain confirmed the deployment of zinc toxicity against MG1655 pGcCzntAp at 8 h post‐infection (p.i.) (Fig. 1A). Re‐imaging the same cells via serial section transmission electron microscopy enabled visualization of intracellular *E. coli*, as well as their correlation with initial confocal images. This analysis revealed that both non‐zinc‐stressed (GFP^+ve^/mCherry^−ve^) and zinc‐stressed (GFP^+ve^/mCherry^+ve^) MG1655 pGcCzntAp reside within membrane‐bound compartments (Fig. 1B). Thus, there is likely to be a role for a member (or members) of the zinc exporter family of transporters in the delivery of antimicrobial zinc from the cytoplasm to these vesicular compartments. Furthermore, by comparison to mCherry^−ve^ MG1655 (Fig. 1B, top inset), mCherry^+ve^ bacteria (Fig. 1B, bottom inset) exhibited a distorted morphology, perhaps reflecting stress and/or membrane perturbation.

### 3.2 Zinc transporter SLC30A1 is constitutively expressed and further upregulated by LPS in human macrophages

**FIGURE 2:**
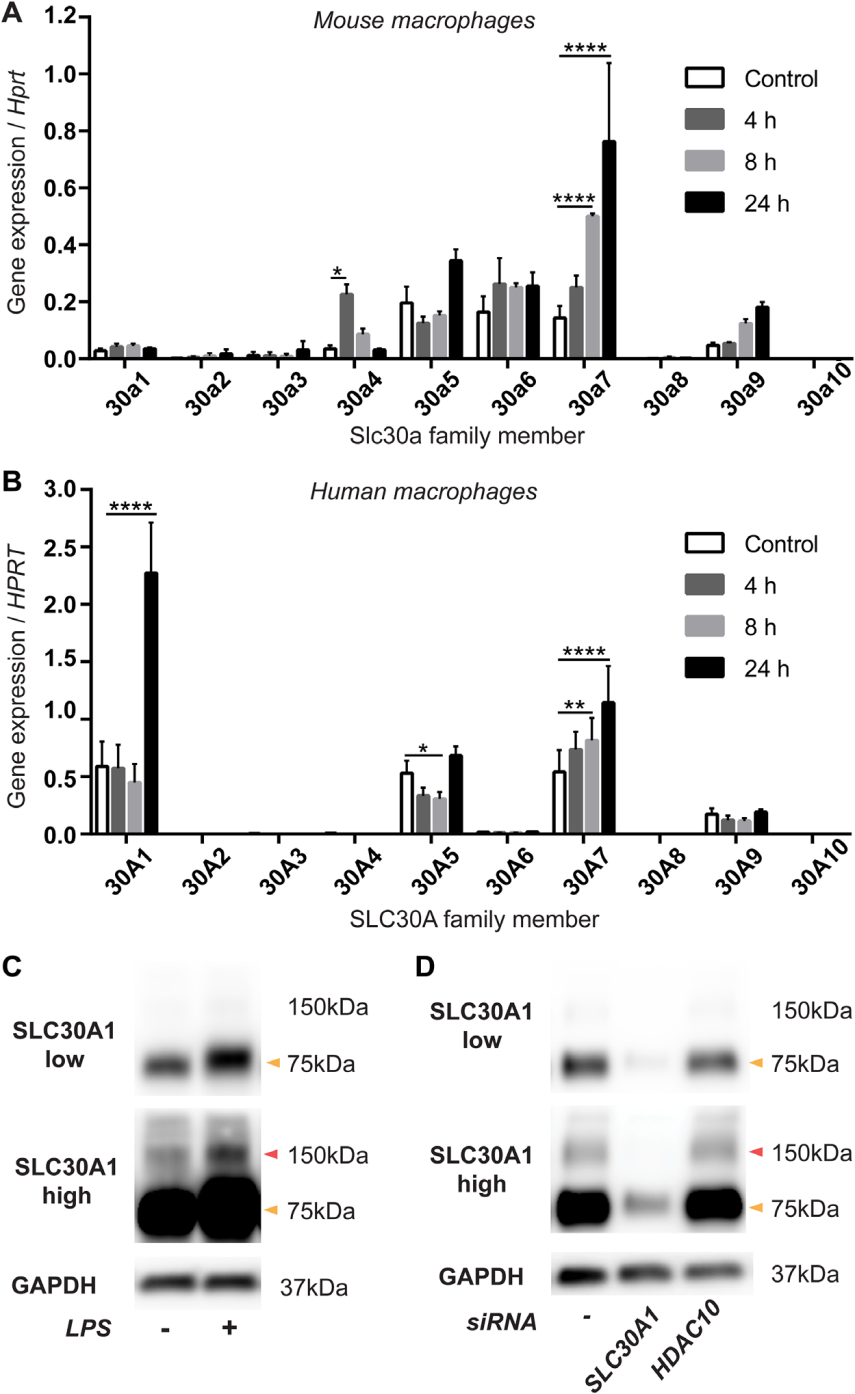
**LPS‐regulated expression of the *Slc30a*/*SLC30A* zinc exporter family in mouse and human macrophages**. (**A**) BMM or (**B**) HMDM were stimulated with LPS for the indicated time points. Expression levels of *Slc30a/SLC30A* family members (relative to *Hprt/HPRT*) were determined by qPCR. (**C** and **D**) HMDM were (**C**) stimulated with LPS or (**D**) transfected with siRNAs against *SLC30A1* or *HDAC10* (control) for 24 h, after which cells were lysed and analyzed by western blot. (**A** and **B**) Data (mean + SEM, *n* = 3) are combined from 3 independent experiments and were analyzed by 2‐way ANOVA with Dunnett's multiple comparisons test. ^*^Denotes *P* < 0.05, ^**^ *P* < 0.01, ^****^ *P* < 0.0001, all other comparisons were not significant. (**C**) and (**D**) depict representative blots from 4 and 3 independent experiments, respectively. Arrows indicate 75 kDa (orange) and 150 kDa (red) protein sizes. High and low exposures of the same SLC30A1 blot are shown in (**C**) and (**D**) to highlight the 150 kDa band

To gain mechanistic insights into the delivery of antimicrobial zinc, we next profiled the LPS‐regulated expression of the family of *Slc30a/SLC30A* zinc transporters in murine BMM (Fig. 2A) and HMDM (Fig. 2B). LPS significantly up‐regulated *Slc30a4* and *Slc30a7* mRNA levels in BMM, with a trend for increased *Slc30a5* and *Slc30a9* expression also observed (Fig. 2A). *Slc30a2*, *Slc30a3*, *Slc30a8*, and *Slc30a10* were all expressed at very low levels in these cells, but LPS treatment also caused a consistent, though non‐significant, elevation in their mRNA expression (data relative to unstimulated control, Supplementary Fig. S1A). In HMDM, LPS significantly increased the expression of *SLC30A1* and *SLC30A7*, with *SLC30A5* mRNA levels being transiently down‐regulated at 8 h post‐stimulation (Fig. 2B). Although absolute mRNA levels were very low, there was also a trend for increased expression of *SLC30A10*, the most closely related SLC30A family member to *SLC30A1* ^29^ (data relative to unstimulated control, Supplementary Fig. S1B). Given that many *Slc30a* transporters were LPS‐inducible at the mRNA level in BMM (Fig. 2A; Supplementary Fig. S1A), while the regulated expression of SLC30A family members was more restricted in HMDM, we subsequently focused on human macrophages. In HMDM, SLC30A1 was particularly highly expressed and LPS‐inducible at both the mRNA (Fig. 2B) and protein level (Fig. 2C). LPS‐inducible SLC30A1 was detected as ∼75 and ∼150 kDa in size in HMDM, as confirmed by gene silencing in these cells (Fig. 2D). This raises the possibility that SLC30A1 in macrophages may exist as a tightly bound multimeric complex and/or glycosylated dimer in addition to a glycosylated monomer.^30^ Similar observations were previously made for the LPS‐inducible expression of the zinc importer SLC39A8 in human macrophages.^19^ We note that the 75 kDa monomer was the most abundantly expressed form of SLC30A1 in HMDM (Fig. 2C, high exposure blot). Interestingly, monomeric SLC30A1 consistently ran at a slightly higher molecular weight in LPS‐treated versus control HMDM, suggestive of an LPS‐inducible post‐translational modification. A previous study demonstrated that *SLC30A1* mRNA levels were increased in human macrophages following *Mycobacterium tuberculosis* infection.^9^ Thus, it is likely that multiple microbial stimuli up‐regulate the expression of this zinc transporter.

### 3.3 Ectopic expression of SLC30A1 in THP‐1 cells drives zinc‐containing vesicle formation

**FIGURE 3:**
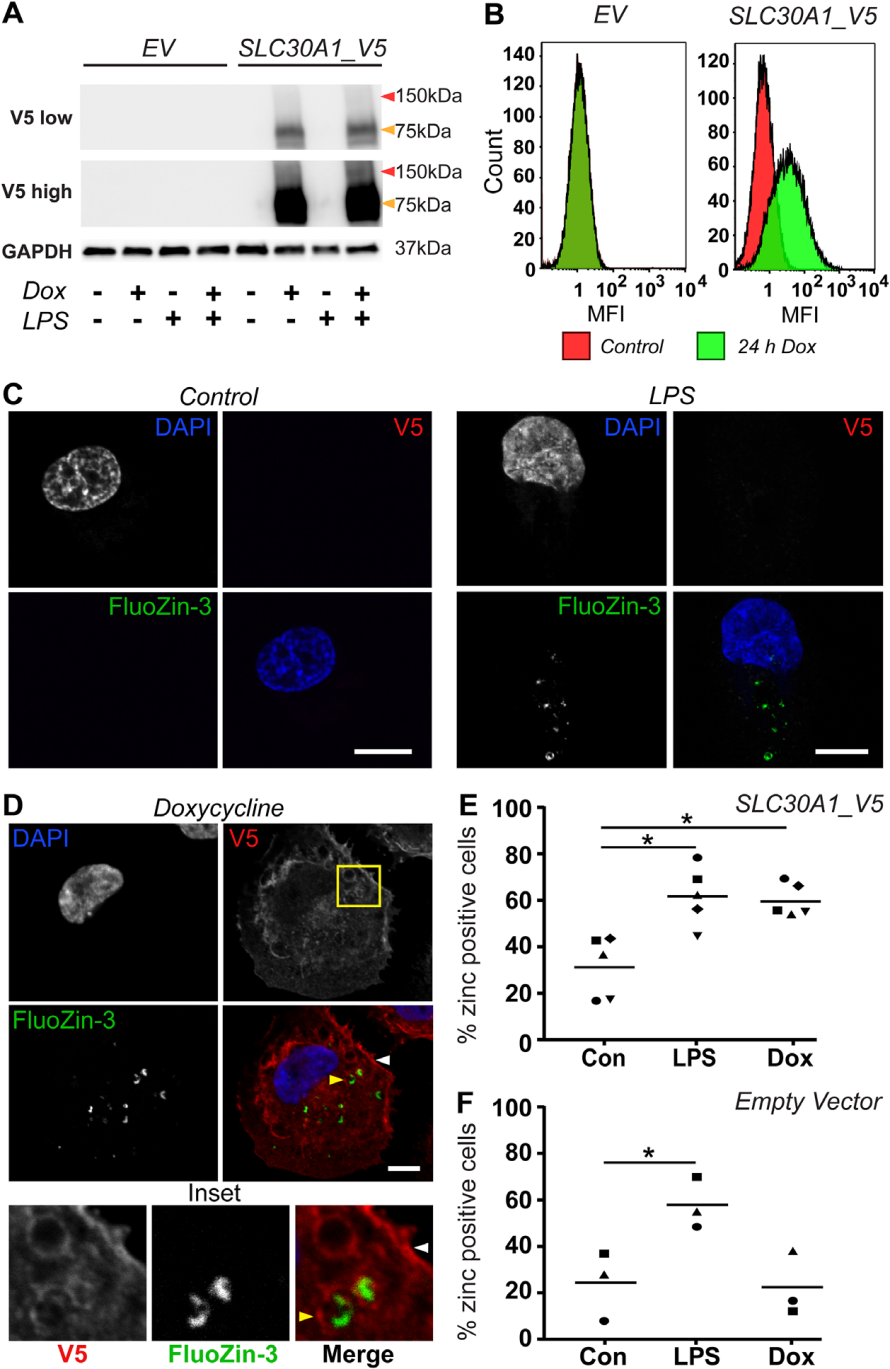
**Ectopically‐expressed SLC30A1_V5 in THP‐1 cells localizes to the plasma membrane and zinc‐containing intracellular compartments**. PMA‐differentiated THP‐1 cells stably transduced with lentivirus expressing either empty vector (EV) or SLC30A1_V5 were left unstimulated (‐, Con) or were stimulated with (**A** and **B**) 100 ng/mL or (C–F) 500 ng/mL doxycycline (Dox) for 24 h. (**A**) Cells were simultaneously stimulated ± 100 ng/ml LPS for 24 h, after which samples were lysed, processed and analyzed by western blot. Blots were probed with Abs against V5 or GAPDH as a loading control. The displayed immunoblots are from a single experiment and are representative of 3 independent experiments. Arrows indicate 75 kDa (orange) and 150 kDa (red) protein sizes. (**B**) Samples were lifted and fixed, before being permeabilized and stained with an anti‐V5 Ab, followed by anti‐mouse Alexa‐647, and analyzed via flow cytometry. Data, which depict histograms from a single experiment, are representative of 2 independent experiments. MFI, median fluorescence intensity. (**C–F**) Cells were simultaneously stimulated ± 100 ng/ml LPS for 24 h, after which cells were fixed in 4% paraformaldehyde, permeabilized and stained sequentially with mouse anti‐V5, anti‐mouse Alexa‐647, DAPI, and FluoZin‐3. (**C** and **D**) Images depict individual SLC30A1_V5 THP‐1 cells from the same experiment and are representative of 5 independent experiments. White arrow indicates plasma membrane, yellow arrow indicates intracellular vesicle. Scale bar represents 10 μm. (**E** and **F**) The percentage of Fluozin‐3 positive THP‐1 cells was determined by blinded quantification of multiple images, with a minimum of 75 cells/experiment counted. Data are combined from 5 (**E**) or 3 (**F**) independent experiments, with the horizontal line depicting mean (data for each experiment have a distinct symbol). Experiments in (**F**) were performed simultaneously to 3 experiments in (**E**), and data in (**E** and **F**) were analyzed by 1‐way ANOVA with Tukey's multiple comparisons test, ^*^ *P* < 0.05

In contrast to observations in HMDM, LPS did not up‐regulate *SLC30A1* mRNA expression in PMA‐differentiated macrophage‐like THP‐1 cells (Supplementary Fig. S2A), despite these cells being LPS‐responsive as assessed by *TNF* expression (Supplementary Fig. S2B). Thus, we used a doxycycline‐inducible system to ectopically express *SLC30A1* in these cells for investigation of its contributions to the macrophage zinc toxicity response. Doxycycline induced *SLC30A1* mRNA expression in THP‐1 cells transduced with a lentiviral construct for SLC30A1, but not empty vector (EV), as expected (Supplementary Fig. S2C). Doxycycline‐inducible epitope‐tagged (V5) SLC30A1 expression in these cells was confirmed by both immunoblotting (Fig. 3A) and flow cytometry (Fig. 3B). In these cells, overexpressed SLC30A1_V5 was detectable at ∼75 kDa (Fig. 3A). However, a weakly expressed higher order complex (∼150 kDa, high exposure blot) was again detected, similar to our observation in HMDM (Fig. 2C).

We next examined the localization of SLC30A1 in macrophages, particularly in relation to LPS‐inducible zinc‐containing vesicles. SLC30A1 has been reported to localize exclusively to the plasma membrane of baby hamster kidney cells,^31^, ^32^ rat astroglial cells,^33^ and rat cortical neurons.^34^ In the absence of ectopic SLC30A1 expression, LPS induced the formation of zinc vesicles in PMA‐differentiated THP‐1 cells, as detected by FluoZin‐3 staining (Fig. 3C). Notably, the staining pattern of these LPS‐inducible zinc vesicles was not as pronounced as we have previously observed in HMDM,^10^, ^12^ being much more sparse and heterogeneous on a population and individual cell level. Upon induction of V5‐tagged SLC30A1 in THP‐1 cells, we observed that SLC30A1 localized to both the plasma membrane and intracellular vesicular structures (Fig. 3D). Interestingly, treatment with doxycycline to induce SLC30A1 expression increased the percentage of FluoZin‐3‐positive THP‐1 cells, with the effect being similar to that observed with LPS stimulation (Fig. 3E). Importantly, doxycycline alone did not have this effect in pLenti_EV‐transduced control cells (Fig. 3F). This suggests that the overexpression of SLC30A1 alone is sufficient to mobilize zinc in THP‐1 cells in a similar fashion to the response triggered by LPS. This hypothesis is further supported by the observation that FluoZin‐3‐stained zinc was often detected within SLC30A1_V5‐positive vesicles (Fig. 3D). We note, however, that this co‐localization was not uniform within a population of macrophages, likely because of the dynamic nature of zinc mobilization.

Although SLC30A1 can prevent cellular zinc toxicity via plasma membrane efflux,^30^, ^31^ examination across a broader range of cell types also supports a contribution to intracellular zinc redistribution. In a human keratinocyte cell line, SLC30A1 localized to the endoplasmic reticulum, nuclear membrane, and Golgi, and was found to regulate the intracellular distribution of zinc, but not the overall cellular concentration.^35^ SLC30A1 was also reported to be expressed at both the plasma membrane and in intracellular compartments in rat mammary glands at different stages of lactation, as well as in mouse mammary epithelial cells.^36^ Indeed, a recent study examining the regulation and modification of SLC30A1 in human pancreatic cancer cells in response to zinc fluctuations concluded that the molecular mechanisms underlying control of zinc homeostasis by SLC30A1 are more complex than previously thought.^30^ Thus, the localization and functions of SLC30A1 appear to be highly dependent on both cell type and the specific cellular signals received.

### 3.4 SLC30A1 subjects engulfed *E. coli* to a zinc stress antimicrobial response in PMA‐differentiated THP‐1 cells

**FIGURE 4:**
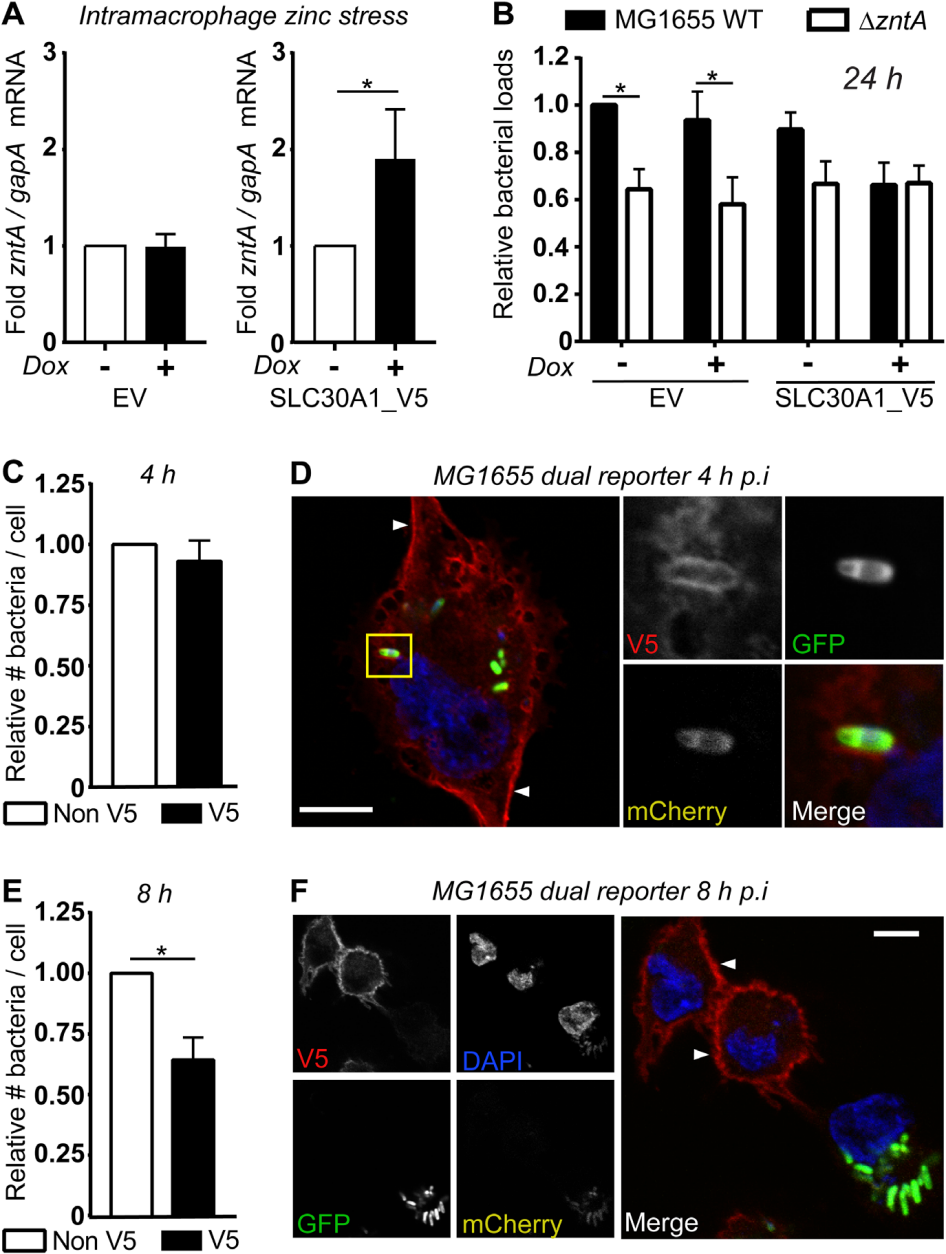
**SLC30A1_V5 promotes a zinc stress response and bacterial killing in THP‐1 cells, and localizes to both the plasma membrane and compartments containing zinc‐stressed *E. coli***. (**A–F**) PMA‐differentiated THP‐1 cells stably transduced with lentivirus expressing either empty vector (EV) or SLC30A1_V5 were left unstimulated (‐) or stimulated with (**A** and **B**) 100 ng/ml or (**C–F**) 500 ng/ml doxycycline (Dox) for 24 h. After washing, THP‐1 cells were infected with (**A**) MG1655 or (**B**) MG1655 and MG1655Δ*zntA* (MO1 100) for 1 h, with gentamicin exclusion used to remove extracellular bacteria. (**A**) At 8 h p.i., cells were lysed, total RNA was isolated, and mRNA levels of *zntA* (relative to the bacterial housekeeping gene *gapA*) determined by qPCR. In each case (EV or SLC30A1_V5), *zntA* mRNA levels in *E. coli* within THP‐1 cells not stimulated with doxycycline were used to calculate the fold change response to enable comparisons across different experiments. (**B**) Cells were lysed and CFU/mL determined at 24 h p.i. For each experiment, data were normalized to the number of wild‐type bacteria within unstimulated EV THP‐1 cells. (**C–F**) SLC30A1_V5 THP‐1 cells were washed and infected with MG1655 pGcCzntAp (MOI 100) for 1 h, using an initial spinfection (5 min at 500 × *g*). Cells were fixed with 4% paraformaldehyde, permeabilized, and then stained with mouse anti‐V5 followed by anti‐mouse Alexa‐647 and DAPI. The average numbers of bacteria within infected cells (V5^−ve^ and V5^+ve^) were determined via blinded quantification of bacterial GFP fluorescence at (**C**) 4 and (**E**) 8 h. Experiments were performed simultaneously, and data were normalized to the number of bacteria within V5^−ve^ cells at each time point. Data are combined from 6 (**A**) or 5 (**B**, **C**, **E**) independent experiments. Data were analyzed by (**A**) Wilcoxon matched‐pairs signed rank test, (**B**) 2‐way ANOVA with Sidak's multiple comparisons test or (**C** and **E**) paired *t*‐test. ^*^ *P* < 0.05, all other comparisons were not significant. (**D** and **F**) Depict individual V5^+ve^ THP‐1 cells infected with MG1655 pGcCzntAp at (**D**) 4 or (**F**) 8 h p.i., representative of 5 independent experiments. Scale bar represents 10 μm, white arrows indicate plasma membrane, yellow box indicates inset displayed on right

We next investigated whether the induction of SLC30A1 increased *zntA* mRNA expression within macrophages. In pLenti_SLC30A1_V5‐transduced THP‐1 cells, doxycycline treatment prior to infection with MG1655 caused an ∼2‐fold increase in *zntA* mRNA at 8 h p.i., with this effect not observed in pLenti_EV‐transduced control cells (Fig. 4A). This is consistent with SLC30A delivering a zinc stress response to intracellular *E. coli* in macrophages. We then assessed if SLC30A1 overexpression affects the intracellular survival of MG1655 within THP‐1 cells. By comparison to wild‐type MG1655, intracellular loads of the zinc‐sensitive MG1655Δ*zntA* mutant that is deficient in the ZntA zinc efflux system^10^, ^12^ were reduced in EV THP‐1 cells in the presence or absence of doxycycline at 24 h p.i. (Fig. 4B; Supplementary Table 3). This confirms that zinc export contributes to the survival of MG1655 in THP‐1 cells, as was previously observed in *E. coli*‐infected HMDM, where *zntA* was both deleted and complemented.^9^ We note, however, that the effect of *zntA* deletion on survival within THP‐1 cells was less pronounced than previously observed for primary human macrophages.^12^ Interestingly, doxycycline‐mediated induction of SLC30A1 expression reduced the intracellular bacterial loads of wild‐type MG1655 to levels similar to those observed with the *zntA* mutant strain. This suggests that the overexpression of SLC30A1 may overcome the resistance of wild‐type MG1655 to the macrophage zinc toxicity response. This effect was not due to a reduction in the initial uptake, as there was no difference in intracellular bacterial loads for either wild‐type MG1655 or MG1655Δ*zntA* from either pLenti_EV‐ or pLenti_SLC30A1‐transduced cells at 2 h p.i. (Supplementary Fig. S2D).

We next utilized the MG1655 zinc stress reporter strain^12^ (Fig. 1), in conjunction with our inducible THP‐1 cell system, to further investigate the capacity of SLC30A1 to elicit an antimicrobial response. As expected, we noted heterogeneity in SLC30A1_V5 expression across the population of pLenti_SLC30A1‐transduced THP‐1 cells after treatment with doxycycline. We took advantage of this heterogeneity, quantifying numbers of MG1655 bacteria within SLC30A1‐positive (V5^+ve^) versus SLC30A1‐negative (V5^−ve^) cells within the same population of THP‐1 cells after doxycycline‐mediated induction of SLC30A1_V5 expression. This revealed that there was no difference in intracellular bacterial loads between V5^+ve^ and V5^−ve^ cells at 4 h p.i (Fig. 4C). At this time point, SLC30A1_V5 localized either predominantly at the plasma membrane or at both the plasma membrane and on intracellular vesicular structures (Fig. 4D) of MG1655‐infected THP‐1 cells. MG1655 found within these SLC30A1_V5‐marked structures were consistently zinc‐stressed (mCherry^+ve^) (Fig. 4D). By 8 h p.i., significantly reduced bacterial loads were observed in V5^+ve^ cells as compared to V5^−ve^ cells (Fig. 4E). At this time point, SLC30A1_V5 predominantly localized to the plasma membrane of cells that did not contain bacteria, with the majority of MG1655 within V5^−ve^ THP‐1 cells found to be only weakly mCherry^+ve^ (Fig. 4F). These data represent the first localization studies of SLC30A1 within macrophages, and, combined with the heightened zinc stress gene signature (Fig. 4A) and enhanced clearance (Fig. 4B, E) of MG1655 in SLC30A1_V5‐expressing THP‐1 cells, strongly support a role for SLC30A1 in the delivery of zinc toward engulfed *E. coli* to initiate a late‐stage zinc toxicity antimicrobial response.

### 3.5 SLC30A1 is not essential for bacterial killing in primary human macrophages

**FIGURE 5:**
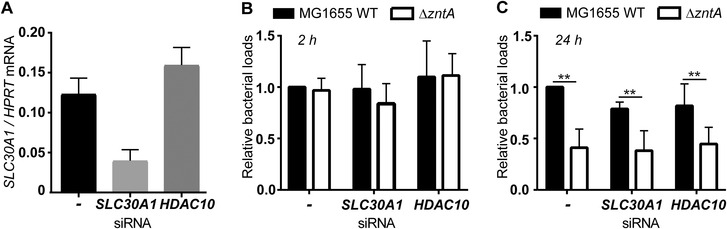
**Targeted genetic knockdown of *SLC30A1* does not prevent the zinc toxicity response**. HMDM were transfected with siRNAs targeting *SLC30A1* or *HDAC10* (control) or with no siRNA (‐) and incubated for 24 h, before undertaking the analyses described in (**A–C**). (**A**) Cells were lysed and total RNA extracted, and mRNA levels of *SLC30A1* (compared to housekeeping gene *HPRT*) determined. Data (mean + range) represent one matched experiment to cells used in (**B**) and are representative of 3 independent experiments. (**B–C**) Cells were infected with either MG1655 or MG1655Δ*zntA* (MOI 100) for 1 h. Extracellular bacteria were then removed by gentamicin exclusion, with cells lysed and CFU determined at (**B**) 2 h and (**C**) 24 h p.i. Fold change was calculated based on the wild‐type MG1655 CFU in HMDM transfected with no siRNA in each experiment. Data (mean + SEM, *n* = 4) are combined from 4 independent experiments. Data were analyzed by 2‐way ANOVA with Sidak's multiple comparisons test, ^**^ *P* < 0.01, all other comparisons were not significant

Somewhat surprisingly, silencing of *SLC30A1* in HMDM (Fig. 5A, see also Fig. 1D) did not have any impact on the clearance of either wild‐type MG1655 or MG1655Δ*zntA* (Fig. 5B and C; Supplementary Table 4). Furthermore, reduced survival of the MG1655Δ*zntA* compared to wild‐type MG1655 at 24 h was still apparent (Fig. 5C), suggesting that the zinc toxicity response was effectively engaged in *SLC30A1*‐silenced HMDM. The lack of effect of *SLC30A1* silencing on bacterial clearance could reflect the complexities of the zinc toxicity pathway (see Section 3.6 below) or be a consequence of the transfection protocol affecting SLC30A1 function in HMDM. It seems more likely, however, that it reflects redundancy in the functions of mammalian zinc exporters in delivering the zinc toxicity response in human macrophages. We note that many SLC30A family members are expressed and regulated in HMDM (Fig. 2B; Supplementary Fig. S1B). Furthermore, LPS still induced zinc‐containing vesicles in PMA‐differentiated THP‐1 cells (Fig. 3C, E, and F), despite the fact that this stimulus did not increase SLC30A1 mRNA in this cell line (Supplementary Fig. S2A). Interestingly, biomolecular fluorescence complementation^37^ in MCF‐7 breast cancer cells reported the homodimerization of SLC30A1, SLC30A2, SLC30A3, SLC30A4, and SLC30A7.^38^ As a homodimer, SLC30A1 localized to the plasma membrane,^38^ however subsequent studies revealed that SLC30A1 also heterodimerized with SLC30A2 and SLC30A4, and that this altered its sub‐cellular localization.^39^ Thus, SLC30A1 may also function in conjunction with other zinc transporters to achieve vesicular delivery of zinc to engulfed bacteria.

### 3.6 Additional factors that may contribute to innate immune‐mediated zinc toxicity

The role of zinc within macrophage antimicrobial responses is likely complex and multi‐layered, extending beyond direct metal ion poisoning. For example, we previously found that reactive oxygen species increased the sensitivity of a uropathogenic *E. coli zntA* mutant to zinc.^12^ This suggests that the zinc toxicity response may cooperate with other innate immune antimicrobial pathways, such as the phagocyte oxidase system. Another innate immune antimicrobial pathway that could intersect with zinc toxicity is TLR‐inducible production of nitric oxide (NO), which is generated by inducible nitric oxide synthase.^1^ In *Salmonella*, NO triggers the mobilization of zinc from a range of zinc metalloproteins, including those involved in sensing nitrosative stress,^40^ DNA binding,^41^ protein synthesis and cell metabolism.^42^ Consequently, effective zinc efflux is required to maintain homeostasis and permit survival of *Salmonella* under conditions of nitrosative stress.^42^ Thus, delivery of zinc to intramacrophage bacteria via the actions of SLC30A1 and/or other transport mechanisms may enhance the antimicrobial effects of NO. We note, however, that our studies were performed in primary human macrophages that generate substantially less NO than primary mouse macrophages upon activation, because of regulatory differences between the human *NOS*2 and mouse *Nos2* genes.^43^ Nonetheless, interplay between innate immune zinc delivery systems and NO may be important during NOS2‐independent nitrosative stress responses in human cells and/or the Nos2‐mediated antimicrobial response in mice.

In summary, the LPS‐inducible zinc exporter SLC30A1 permits mobilization of zinc toward intracellular *E. coli* and the generation of a zinc‐mediated antimicrobial response in macrophages. On the basis of studies on the *E. coli* homologue, YiiP, it is likely that SLC30A1 requires a proton gradient to deliver zinc in a pH‐driven, sodium‐independent, and calcium‐sensitive manner (1:1, Zn^2+^/H^+^).^44^ In the context of macrophage infection, acidification of the phagolysosome by vacuolar ATPases^45^ might be predicted to subsequently permit SLC30A1‐mediated delivery of zinc into phagolysosomal compartments from the cytoplasm. This could provide a means of temporal diversification of macrophage antimicrobial responses. At this stage, our understanding of the processes regulating the formation and/or stability of the zinc‐containing compartment remain quite rudimentary, but further characterisation of the SLC30A1^+ve^ zinc‐containing compartment should generate new insights into these processes. In the longer term, our findings may lead to new avenues for manipulating SLC30A1 expression or function to enhance innate immune‐mediated zinc poisoning as an anti‐infective strategy.

## AUTHORSHIP

C.J.S., J.v.P., J.B.C., and J.R. performed experiments, with assistance from R.K. and M.D.P.; D.F. and N.J.B. performed key initial studies and contributed to project design; K.M.P. and T.K. provided essential reagents (bacterial strains and anti‐SLC30A1 Ab, respectively); M.J.S., R.K., M.A.S., and R.G.P. designed and supervised the project; and C.J.S., J.v.P., R.K., and M.J.S. wrote the manuscript. All authors read and approved the final manuscript.

## DISCLOSURE

The authors declare no conflict of interest.

## Supporting information

Supporting Information

Supporting Information

Supporting Information

Supporting Information

Supporting Information

Supporting Information
