# Supplementary material for: Frontline Science: LPS‐inducible SLC30A1 drives human macrophage‐mediated zinc toxicity against intracellular Escherichia coli
Source: J Leukoc Biol. 2020 May 22;109(2):287–97. doi: 10.1002/JLB.2HI0420-160R (PMC7891337; doi:10.1002/JLB.2HI0420-160R)
Supplement: Supplementary file 1 — Supporting Information [file JLB-109-287-s001.docx]

**Supplementary Material**

*Bacterial strains and culture conditions*

Bacterial strains and plasmids used in this study are listed in Supplementary Table 5. All *E. coli* strains were routinely cultured at 37°C on solid or in Lysogeny Broth (LB) medium supplemented with appropriate antibiotics. The Δ*zntA* mutant of MG1655 used in this study has previously been described^12^.

*Correlative light-electron microscopy*

Cells grown on 35 mm gridded MatTek dishes were imaged live on a Nikon Inverted Microscope stand with Andor Dragonfly Spinning disc Scanhead. Cells were then washed with PBS and fixed in 2.5% glutaraldehyde for 30 min. Dishes were washed again in PBS then processed for electron microscopy as previously described^46^. Areas of interest were identified using alphanumeric code imprinted into resin from the gridded MatTek dish and ultrathin sections were cut on an ultramicrotome (UC6: Leica). Micrographs were acquired on a JEOL1011 transmission electron microscope operating at 80kV and correlated with light images using Photoshop (Adobe Inc).

*LPS stimulation*

Where indicated, HMDM or PMA-differentiated THP-1 cells were stimulated with 100 ng/mL purified *Salmonella enterica* serotype Minnesota LPS (Sigma-Aldrich).

*Infection assays*

Bacterial infection assays were performed as previously described^47^. In brief, bacteria were grown overnight at 37°C in LB medium and then used to infect cultured macrophages at MOI 100. After 1 h, media was removed and replaced with media containing 200 µg/mL gentamicin for 1 h, before further culture in media containing 20 µg/mL gentamicin. To determine intracellular bacterial loads, cells were lysed with 0.1% Triton X-100 in PBS at and plated in triplicate onto LB agar. For confocal microscopy, a 5 min, 500 G spin at 37°C was utilised during infection of HMDM or PMA-differentiated THP-1 cells with *E. coli* to enhance the number of bacteria taken up into host cells.

*Lentiviral construct generation*

Expression plasmid pLenti_EV was modified through the insertion of a multiple cloning site (MCS) to generate pLenti_MCS (Empty vector). SLC30A1_V5 was then PCR amplified from a mammalian expression construct (pEF6_SLC30A1), and sub-cloned to create pLenti_SLC30A1_V5. Primers used to generate lentiviral plasmid constructs are detailed in Supplementary Table 1.

*Immunoblotting*

Western blotting was used to examine protein expression of specific host cell zinc transporters, as previously described^48^. Antibody concentrations are listed in Supplementary Table 2. Enhanced chemiluminescent substrate (Bio-Rad) was added to the blot, with the image visualised on an Amersham Imager 600.

*Flow cytometry*

For staining of V5-tagged SLC30A1 on THP-1 cells, cells were washed twice with PBS, before being resuspended in 450 µL of Lift buffer (PBS containing 0.1% sodium azide [Sigma-Aldrich] and 1 mM EDTA [Gibco]) and fixed with 1% formaldehyde (Sigma-Aldrich). Cells were then washed and permeabilised with PERM buffer (1% FBS, 0.25% Saponin [Sigma-Aldrich], 5 mM EDTA in PBS). Non-specific antigens were blocked via Trustain FcX (anti-Fc receptor, BioLegend), followed by staining with anti-V5 and anti-mouse Alexa-647 antibodies, respectively (for details see Supplementary Table 2). Cells were resuspended in 300 µL PBS and analysed on a Gallios Flow Cytometer. Gating was utilised to exclude cellular debris and cell aggregates. A 638 nm (red) laser was utilised to excite Alexa-647, with data analysed by Kaluza Analysis 1.3.

*Quantitative real-time PCR (qPCR)*

For analysis of bacterial gene signatures, infected cells were lysed in TRIzol (Invitrogen) and total RNA was extracted using Direct-zol RNA Miniprep Kit (Zymo), following the manufacturer’s instructions. Analysis of bacterial gene expression within macrophages was performed as previously described^12^. For analysis of host cell zinc transporters, HMDM, BMM or PMA-differentiated THP-1 cells were stimulated with LPS for the indicated time points or were left untreated. Cells were then lysed, RNA extracted and DNAse treated utilising the Qiagen (Germany) RNeasy Mini Kit. One µg of RNA was reverse transcribed to cDNA using Oligo dT (Invitrogen). Levels of bacterial *zntA* mRNA (relative to the *E. coli* housekeeping gene *gapA*), human *SLC30A1-A10* (relative to housekeeping gene *HPRT*), or mouse *Slc30a1-30a10* (relative to housekeeping gene *Hprt*) mRNA were quantified by qRT-PCR utilising the Applied Biosystems 7900HT fast RT-PCR system and the ΔC_t_ method. Primers used for qRT-PCR (Sigma, Australia) are listed in Supplementary Table 6.

*Selective gene silencing*

Three combined sets of siRNA (Life Technologies, see Supplementary Table 7) were added to a final concentration of 1 μM to HMDM within a 0.4 cm gap Gene Pulserâ /MicroPulser electroporation cuvette (Bio-Rad). These were electroporated at 260 V and 1000 μF capacitance using a Bio-Rad electroporator. Cells were recovered at room temperature for 10 min, washed and plated according to experimental requirements, with gene silencing allowed to proceed for 24 h before subsequent experimentation.
