# Supplementary material for: Frontline Science: LPS‐inducible SLC30A1 drives human macrophage‐mediated zinc toxicity against intracellular Escherichia coli
Source: J Leukoc Biol. 2020 May 22;109(2):287–97. doi: 10.1002/JLB.2HI0420-160R (PMC7891337; doi:10.1002/JLB.2HI0420-160R)
Supplement: Supplementary file 2 — Supporting Information [file JLB-109-287-s002.docx]

**
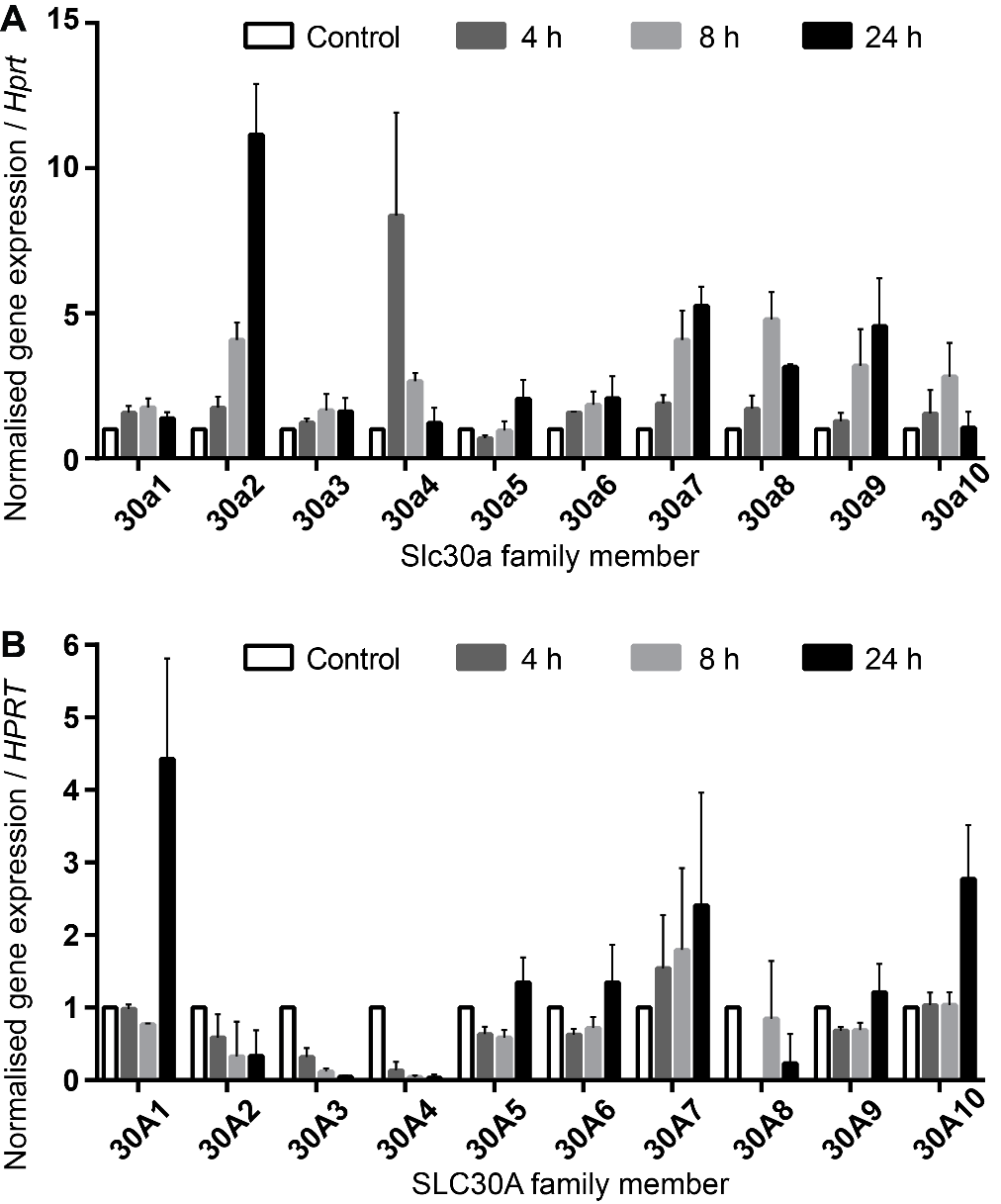
**

**Supplementary Figure 1. Regulated expression of** *SLC30A* family members in macrophages**.** (A) BMM or (B) HMDM were stimulated with LPS, with samples lysed and prepared for mRNA analysis at 4, 8 and 24 h post-LPS stimulation. Expression levels across the *Slc30a*/*SLC30A* family were compared to *Hprt/HPRT*. Values were then normalised to control expression levels. Data (n=3, mean + SEM) in both cases are combined from 3 independent experiments.
