# Supplementary material for: Frontline Science: LPS‐inducible SLC30A1 drives human macrophage‐mediated zinc toxicity against intracellular Escherichia coli
Source: J Leukoc Biol. 2020 May 22;109(2):287–97. doi: 10.1002/JLB.2HI0420-160R (PMC7891337; doi:10.1002/JLB.2HI0420-160R)
Supplement: Supplementary file 3 — Supporting Information [file JLB-109-287-s003.docx]

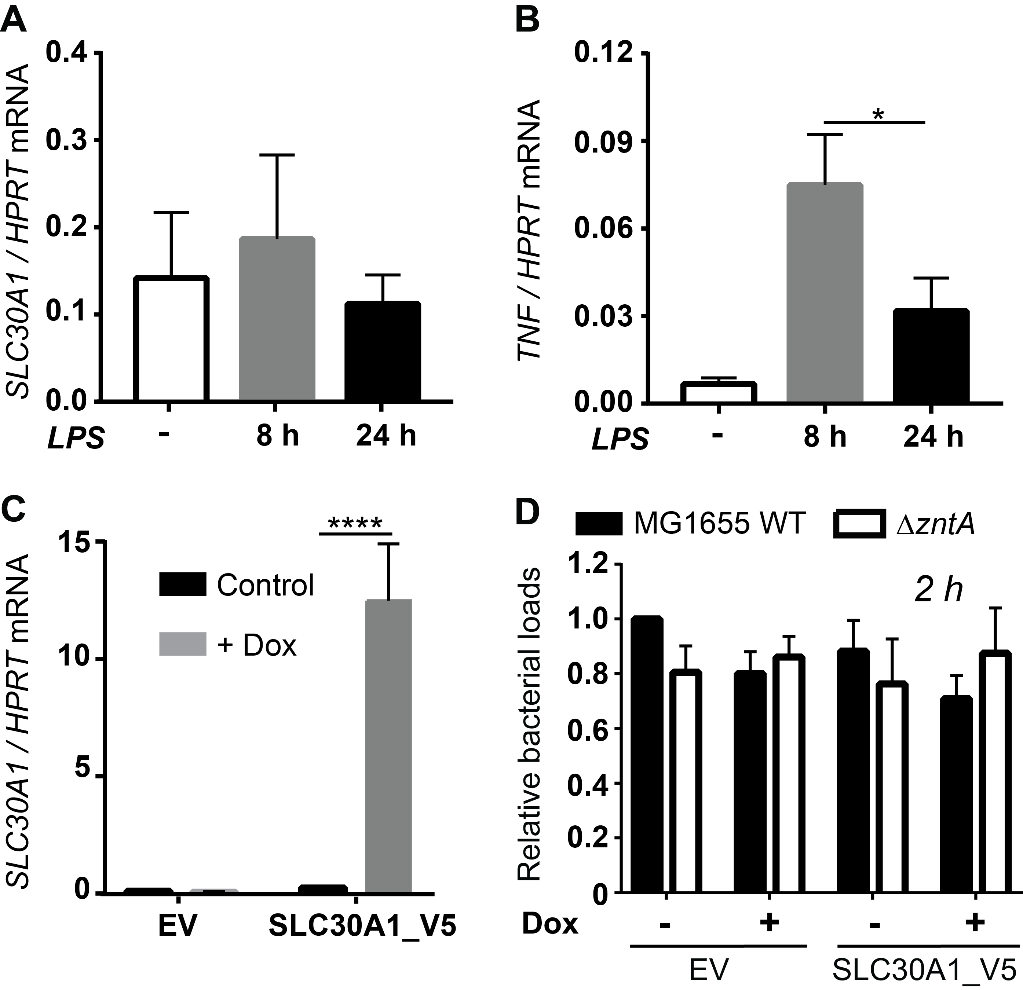


Supplementary Figure 2. Regulated gene expression and bacterial clearance in THP-1 cells**. (A, B)** Lentivirally-transduced Empty Vector (EV) THP-1 cells were PMA differentiated before being stimulated with 100 ng/mL LPS for 24 h. Cells were then lysed, RNA extracted and cDNA prepared for qPCR. Levels of **(A)** *SLC30A1* or **(B)** *TNF* mRNA were compared to levels of *HPRT*. **(C, D)** Lentivirally-transduced THP-1 cells (EV or SLC30A1_V5) were PMA differentiated before being left unstimulated (-) or stimulated with 100 ng/mL doxycycline (Dox) for 24 h. **(C)** Cells were then prepared as above for qPCR, with levels of *SLC30A1* mRNA being compared to *HPRT*. **(D)** After washing, cells were infected with MG1655 or MG1655Δ*zntA* (MOI 100) for 1 h, followed by gentamicin exclusion to remove extracellular bacteria. Cells were lysed and CFU/mL determined at 2 h. Fold change was calculated based on the number of wild type bacteria within unstimulated EV THP-1 cells. Data **(A, B, C)** (mean + SEM, n=3) are from 3 independent experiments and were analysed by **(A, B)** One-Way ANOVA with Dunnett’s multiple comparisons test or **(C)** Two-Way ANOVA with Sidak’s multiple comparisons test. Data **(D)** (mean + SEM, n=5) are from 5 independent experiments and were analysed by Two-Way ANOVA with Sidak’s multiple comparisons test. * denotes p<0.5, **** p<0.0001, all other comparisons not significant.
