# Supplementary material for: Frontline Science: LPS‐inducible SLC30A1 drives human macrophage‐mediated zinc toxicity against intracellular Escherichia coli
Source: J Leukoc Biol. 2020 May 22;109(2):287–97. doi: 10.1002/JLB.2HI0420-160R (PMC7891337; doi:10.1002/JLB.2HI0420-160R)
Supplement: Supplementary file 4 — Supporting Information [file JLB-109-287-s004.docx]

##### **Supplementary Table 1. List of primers used for generation of lentiviral constructs**

| *Primer* | *Sequence (5’-3’)* | *Purpose* | *Size (bp)* |
| --- | --- | --- | --- |
| Fw Lenti MCS | GATCCTGTACAAGCGCTCTCGAGCCTGCAGGACTCGAGCCTCGAGGTAACTTCGAAG | Used to amplify a MCS containing BamHI and EcoRI sites | 61 |
| Rv Lenti MCS | AATTCTTCGAAGTTACCTCGAGGCTCGAGTCCTGCAGGCTCGAGAGCGCTTGTACAG |  |  |
| SLC30A1_BsrGI | ATCGATGTACATCTGCTCCTCCTGTTGATA | Used to amplify SLC30A1_V5 fragment, including flanking BsrGI and NheI sites | 1770 |
| SLC30A1_V5NheI | CAGCTGCTAGCAACAACAGATGGCTGGCA |  |  |
| Lenti Fw | GAAGGTGGAGAGAGAGACAGAG | Screening primers for PCR confirmation and sequencing of constructs | MCS = 914  SLC30A1_V5 = 2608 |
| Lenti Rv | CTAGGGGAGGAGTAGAAGGT |  |  |
| Lenti 30A1 Centre 1 | CTCTGCCTCTTCCACCATCA | Internal primers used for sequence confirmation (to ensure full coverage of inserted fragments) |  |
| Lenti 30A1 Centre 2 | GTGTGAACTTGCCTGCAGAA |  |  |
| Lenti Centre seq | CCAACTTTCCGTACCACTT |  |  |

**Supplementary Table 2. List of antibodies used for immunoblotting (IB), immunofluorescence (IF) or flow cytometry (FC)**

| *Specificity* | *Description* | *Use* | *Dilution*  *(concentration)* | *Source/Reference* |
| --- | --- | --- | --- | --- |
| V5 epitope tag | Mouse monoclonal anti-V5 | IB  IF  FC | 1:1000  1:1000  1:1000  (1 µg/mL) | Serotec, Oxford, UK |
| GAPDH | Rabbit monoclonal anti-GAPDH | IB | 1:2500  (0.1 µg/mL) | Cell Signalling Technology, Boston, USA |
| SLC30A1 | Mouse monoclonal anti-SLC30A1 | IB | 1:3000 | ^30^ |
| Mouse-IgG | Horse anti-mouse IgG HRP | IB | 1:2500  (0.4 µg/mL) | Cell Signalling Technology |
| Mouse-IgG | Chicken anti-mouse IgG Alexa647 | IF | 1:1000  (2 µg/mL) | Invitrogen |
| Human Fc-receptor | Human anti-Fc receptor | FC | (0.5 µg/million cells) | Biolegend |
