# Supplementary material for: Frontline Science: LPS‐inducible SLC30A1 drives human macrophage‐mediated zinc toxicity against intracellular Escherichia coli
Source: J Leukoc Biol. 2020 May 22;109(2):287–97. doi: 10.1002/JLB.2HI0420-160R (PMC7891337; doi:10.1002/JLB.2HI0420-160R)
Supplement: Supplementary file 5 — Supporting Information [file JLB-109-287-s005.docx]

##### Supplementary Table 3. Average CFU values (n=5 experiments) of intracellular *E. coli* loads in lentivirally-transduced THP-1 cells (corresponding to Fig. 4B and Fig. S2D)

|  | MG1655 | | | | MG1655 ∆*zntA* | | | |
| --- | --- | --- | --- | --- | --- | --- | --- | --- |
|  | **EV** | | **SLC30A1_V5** | | **EV** | | **SLC30A1_V5** | |
| **Dox** | **-** | **+** | **-** | **+** | **-** | **+** | **-** | **+** |
| *2 h* | 32540 | 25800 | 28860 | 23200 | 26100 | 27920 | 25000 | 28694 |
|  |  |  |  |  |  |  |  |  |
| *24 h* | 6834 | 6542 | 6480 | 5046 | 4721.4 | 4623.4 | 5040 | 4898 |

##### Supplementary Table 4. Average CFU values (n=4 experiments) of intracellular *E. coli* loads following siRNA knockdown in HMDM (corresponding to Fig. 5B-C)

|  | MG1655 | | | MG1655 ∆*zntA* | | |
| --- | --- | --- | --- | --- | --- | --- |
| **siRNA** | *-* | *SLC30A1* | *HDAC10* | *-* | *SLC30A1* | *HDAC10* |
| *2 h* | 30333.33 | 24733.33 | 27880.56 | 28647.22 | 22444.44 | 30058.33 |
|  |  |  |  |  |  |  |
| *24 h* | 11805.56 | 8797.222 | 9800 | 1654.639 | 1152.861 | 1757.583 |

##### Supplementary Table 5. *E. coli s*trains and/or plasmids utilised in this study

| *Name* | *Description* | *Source/Reference* |
| --- | --- | --- |
| MG1655 | *E. coli* K-12 strain MG1655 | ^49^ |
| pGcCzntAp | Plasmid encoding constitutively-expressed GFP and zinc-inducible mCherry. Contains promoter of *zntA* upstream of mCherry gene. | ^12^ |
