# Supplementary material for: Frontline Science: LPS‐inducible SLC30A1 drives human macrophage‐mediated zinc toxicity against intracellular Escherichia coli
Source: J Leukoc Biol. 2020 May 22;109(2):287–97. doi: 10.1002/JLB.2HI0420-160R (PMC7891337; doi:10.1002/JLB.2HI0420-160R)
Supplement: Supplementary file 6 — Supporting Information [file JLB-109-287-s006.docx]

##### Supplementary Table 6. List of primers utilised for qPCR analysis

| *Primer name* | *Sequence (5’-3’)* |
| --- | --- |
| *E.coli*_ZntA_Fw  *E.coli*_ZntA_Rv | CGAAGCACAGGTTGCTGAAC  CGCAAATCAATACGCGCTCA |
| *E.coli*_GAPA_Fw  *E.coli*_GAPA_Rv | GGTGCGAAGAAAGTGGTTATGAC  GGCCAGCATATTTGTCGAAGTTAG |
| *hSLC30A1* RT-Fwd  *hSLC30A1* RT-Rev | AAATTGGACCCCGCAGACCC GCCCTATCTTCTTCCAGTTCCAT |
| *hSLC30A2* RT-Fwd  *hSLC30A2* RT-Rev | CACTGTGACCCCAAGAAGGG  GCTCCCAAGATCTCAACGACT |
| *hSLC30A3* RT-Fwd  *hSLC30A3* RT-Rev | TGCACAGCGACTACCACATC  GCAGCACAAAGGCCATTAACA |
| *hSLC30A4* RT-Fwd  *hSLC30A4* RT-Rev | AAGCGCCATCATACTCACCC  GCTGACAAAACCTCTAAGCGA |
| *hSLC30A5* RT-Fwd  *hSLC30A5* RT-Rev | GTGGACCACTAAGGACTTTGC  CTCCCCTTGTCTTTGCTGGT |
| *hSLC30A6* RT-Fwd  *hSLC30A6* RT-Rev | AGCTGTGCAGCTCCTTATCA  CTTCCAGGACCTTCGGTCAG |
| *hSLC30A7* RT-Fwd  *hSLC30A7* RT-Rev | TACTCAGGCTGGAGTGAGACA  TCTTACTGGCAGAAAAATTTGGTCC |
| *hSLC30A8* RT-Fwd  *hSLC30A8* RT-Rev | CCTGGCCGTCATGGAGTTT  TGTTGGAGTTCCACACTTTCT |
| *hSLC30A9* RT-Fwd  *hSLC30A9* RT-Rev | CCATGGAGTCATGGGATTGCT  GCAACAAGAAGTGTTGCTCCT |
| *hSLC30A10* RT-Fwd  *hSLC30A10* RT-Rev | TCGCAAACGTAGCAGGTGAT  CCCATCACATGCAAAAGTACACC |
| *hHPRT*_F  *hHPRT*_R | TCAGGCAGTATAATCCAAAGATGGT  AGTCTGGCTTATATCCAACACTTCG |
| *mSlc30a1* RT-Fwd  *mSlc30a1* RT-Rev | CAGGCAGAGCCAGAAAAATTGAGAA  GCCCTGTTGTCTTCCGCTTC |
| *mSlc30a2* RT-Fwd  *mSlc30a2* RT-Rev | TGCGAGCTGCCATCGTCC  GATCCTAAGAAGGACCGGGCTC |
| *mSlc30a3* RT-Fwd  *mSlc30a3* RT-Rev | GGCTATTGACTCCACGGCTGA  TCAGGCTGGTATTGCTCCACC |
| *mSlc30a4* RT-Fwd  *mSlc30a4* RT-Rev | TGATCGGAGAGCTTGTAGGTGGA  GACAGCCACAAAGCAAGCAGAG |
| *mSlc30a5* RT-Fwd  *mSlc30a5* RT-Rev | CAAAGACAAGAGGGGCTGCT  CCGGGTGTTCAGCCATCTTT |
| *mSlc30a6* RT-Fwd  *mSlc30a6* RT-Rev | TGGCTCATTGGCTGGGTCAG  ACAGTCAGCGTGGACACCAG |
| *mSlc30a7* RT-Fwd  *mSlc30a7* RT-Rev | CGGGTGGAGAAGATGTTGCC  TTGTCCGACAGGATGGACCT |
| *mSlc30a8* RT-Fwd  *mSlc30a8* RT-Rev | TTTGGGTGGTATCGAGCAGAGAT  GGTACAGCAGCACACCAGTC |
| *mSlc30a9* RT-Fwd  *mSlc30a9* RT-Rev | CAAGACCCTCTCCAAGTAAGAGTCA  AGGCAGAACTCGTTTATTGCTCTC |
| *mSlc30a10* RT-Fwd  *mSlc30a10* RT-Rev | GGGTCAACATGGAAGAGCTGATG  TTCTGGTGTTTGATGTGCAAGGT |
| *mHprt*_F  *mHprt*_R | GCAGTACAGCCCCAAAATGG  AACAAAGTCTGGCCTGTATCCAA |

**Supplementary Table 7. Sense and antisense sequences for silencing RNAs**

|  | *SLC30A1* | *HDAC10* |
| --- | --- | --- |
| *Set 1* | CCCUGCAAAGCAUUUGUAGAAAUAA  UUAUUUCUACAAAUGCUUUGCAGGG | CGGAGUCAGUGUGCAUGACAGUACA  UGUACUGUCAUGCACACUGACUCCG |
| *Set 2* | CCCUGCUGUUGUGAUAGAGAUUAAA  UUUAAUCUCUAUCACAACAGCAGGG | UCACUGCACUUGGGAAGCUCCUGUA  UACAGGAGCUUCCCAAGUGCAGUGA |
| *Set 3* | GGAAGUACAAGUGAAUGGAAAUCUU  AAGAUUUCCAUUCACUUGUACUUCC | GGUGGUUUCCUGAGCUGCAUCUUGG  CCAAGAUGCAGCUCAGGAAACCACC |
